# Dynamic Olfactometry and Oil Refinery Odour Samples: Application of a New Method for Occupational Risk Assessment

**DOI:** 10.3390/toxics10050202

**Published:** 2022-04-20

**Authors:** Andrea Spinazzè, Elisa Polvara, Andrea Cattaneo, Marzio Invernizzi, Domenico Maria Cavallo, Selena Sironi

**Affiliations:** 1 Department of Science and High Technology DiSAT, Università degli Studi dell’Insubria, Via Valleggio 11, 22100 Como, Italy; andrea.spinazze@uninsubria.it (A.S.); andrea.cattaneo@uninsubria.it (A.C.); domenico.cavallo@uninsubria.it (D.M.C.); 2 Politecnico di Milano, Department of Chemistry, Materials and Chemical Engineering “Giulio Natta”, Piazza Leonardo da Vinci 32, 20133 Milano, Italy; marzio.invernizzi@polimi.it (M.I.); selena.sironi@polimi.it (S.S.)

**Keywords:** VOC, refinery emissions, olfactometry, occupational health, olfactometric panel, odorous emissions

## Abstract

Refineries are characterized by relevant odour impacts, and the control and monitoring of this pollutant have become increasingly important. Dynamic olfactometry, a sensorial analysis that involves human examiners, is currently the most common technique to obtain odour quantification. However, due to the potential presence of hazardous pollutants, the conduction of occupational risk assessment is necessary to guarantee examiners’ safety. Nevertheless, the occupational risk for olfactometric examiners, specifically correlated with oil refineries emissions, has not been investigated yet. Therefore, this paper applies a new methodology of risk assessment for workers involved in dynamic olfactometry, focusing on odorous refineries emissions. The chemical characterization of refinery emissions was obtained by TD-GC-MS, analysing odorous samples collected at different refinery odour sources. A database of chemical pollutants emitted from a refinery plant was built up, and the minimum dilution values to be adopted during the analysis of refinery odorous samples was calculated. In particular, this evaluation highlighted that, in this scenario, a non-negligible carcinogenic risk may exist for panellists exposed to refineries’ samples, and the carcinogenic risk is sometimes higher than what is acceptable. Therefore, a minimum dilution value between 1.01 and 5, according to the specific sample, must be set to guarantee the examiners’ safety.

## 1. Introduction

Environmental odour is an increasing environmental problem involving several industrial plants [1,2,3]. Refineries are one of the categories most affected by the emission of odorous compounds, in particular volatile organic compounds (VOCs) and sulphur compounds, arising mainly from the operative process, the storage tanks, the transport pipelines, and the waste areas [4,5,6,7,8,9]. Odour nuisance often raises concerns among the population living near these plants due to the common belief that malodorous emissions may negatively affect human health [10,11]. Driven by this, regulatory agencies are progressively requiring refineries to monitor and reduce their odour emissions. In addition, an increasing number of countries have introduced specific regulations regarding odour pollution [12,13]. To study and characterize odorous emissions, several techniques have been developed. One of the most diffuse, and the only technique standardized at the European level to quantify odour concentration, is dynamic olfactometry. This method, standardised by EN 13725:2022 [14], allows the measurement of a sample’s odour concentration, expressed in European odour units per cubic meter (ou_E_/m^3^), by dilution of the odorous sample with neutral air via specific equipment called an olfactometer. Dynamic olfactometry is a sensorial analysis which exploits the human nose as a sensor, able to perceive and distinguish the presence of an odour, and therefore, directly involves human assessors (also called panellists). During the analysis, the odorous sample is diluted with neutral air and presented to panellists at increasing concentrations. Therefore, during the analysis, panellists directly sniff the emission samples and the hazardous compounds potentially contained in them, even diluted. In addition, according to the standard’s sampling requirement, examiners are exposed to odorous mixtures collected directly at the odour source, and therefore, they can be exposed to significant concentrations of toxic pollutants. For these reasons, panellists involved in olfactometric analysis are exposed to undefined occupational exposure risk. Despite the issue of the toxicological risk associated with odour emissions for citizens and workers being discussed in several scientific papers [15,16] (both on citizens and workers), the specific risk assessment for olfactometric workers has not yet been sufficiently investigated and assessed. Indeed, in the literature, only a limited number of studies have dealt with the exposure risk of olfactometric workers, and many of these refer to literature data [11,17,18] or emission limit values [19]. The exposure risk assessment for these examiners correlated with the analysis of real odorous samples appears, therefore, fundamental to define appropriate actions to protect their health, and thus, to safely conduct olfactometric analyses. It should be noted that, due to the technical requirements of this kind of analysis, no classical protective measurement can be adopted to ensure the reduction of exposure of the panellists involved in dynamic olfactometry: for example, respiratory personal protective equipment cannot be employed according to the standard’s requirements (EN 13725:2022). In addition, the standard does not allow the removal of hazardous pollutants to avoid sample alteration. Therefore, at the current state, the evaluation of a minimum dilution value appears the only useful risk management measure to guarantee panellists’ safety: due to odorous emissions being presented to examiners at increasing concentrations, a valuable protection measure to guarantee panellists’ safety is the definition of the minimum dilution step not to be exceeded during olfactometric analysis [19]. This parameter can be assessed by estimating the exposure risk evaluated for the tested, as-is, odorous sample, and comparing it with a minimum dilution level to be adopted (e.g., the dilution step at which to stop the analysis) to ensure panel safety (i.e., keep the risk at an acceptable level). The dilution steps usually available in olfactometers are 14, varying between 1:4 and 1:65,000. By keeping the dilution ratio of the airflow sent by the olfactometer to the human assessors over the determined minimum dilution ratio, we can assume that the panels are exposed to an admissible risk. 

Due to the relevance of their emissions, it is worth focusing on samples of gaseous emissions from oil refineries [20,21,22]. Indeed, the oil refining industry, at the European level, is responsible for about 2% of the overall VOCs emission generated by industrial activities [23]. In addition, it is well known that some compounds belonging to this chemical class (e.g., “BTEX”: benzene, toluene, ethylbenzene, xylenes) have several negative effects on human health [24,25,26,27,28]. For these reasons, the occupational exposure risk for olfactometric examiners became fundamental during the analysis of odorous samples, in particular for those collected at refinery plants. Despite the potential health effects of exposure to refinery pollutants, to the best of our knowledge, in the scientific literature, studies and papers analysing refinery emissions collected directly from the emission source, as prescribed by the standard, are not available. In this work, an experimental analysis of odour emissions collected in refinery plants was conducted, to evaluate the health risk for panellists involved in odour quantification by dynamic olfactometry. The novel aspect of this research is the creation of a database of volatile organic pollutants emitted by refinery plants, and the estimation of the toxicological risks for panellists involved in dynamic olfactometry. To assess this occupational risk and evaluate a minimum dilution value to be applied during the analysis of refinery odorous samples, a standardized method is not yet defined. Indeed, the standard does not describe an analytical procedure to evaluate this risk, but only recommends the use of current relevant exposure limits. However, this prescription still appears too general, and, at the current state, a detailed and standardized procedure is not yet available. On the other hand, in the scientific literature, some articles have proposed a method for assessing the exposure risk for panellists [11,17,18,19]. However, these studies apply, in absence of a standardized method, different occupational exposure limit (OEL) values, obtaining different results [29]. In addition, these studies do not describe how to consider, in the toxicological evaluation, the compounds observed for which there is no exposure limit in the proposed databases. This problem in the presence of a high number of undefined compounds in a real odorous sample can imply an underestimation of the real risk [29]. Therefore, this paper aims to apply a new method, defined by the authors [30], that considers all of the critical aspects observed in the available literature [29], to establish the occupational risk for panellists involved in olfactometric analyses, and, to prevent a potentially dangerous exposure to chemicals of the olfactometric panellists. In addition, it is the first attempt to evaluate the minimum dilution values to be adopted during the analysis of real odorous samples collected at different odour sources of a refinery plant.

## 2. Materials and Methods

### 2.1. Sampling 

To evaluate the minimum dilution value for refinery emissions, a sampling campaign was conducted, during which 18 samples were collected at the plant’s main emission sources.

According to EN 13725 prescription, odorous emissions were sampled using Nalophan^TM^ bags equipped with a Teflon^TM^ inlet tube. During sampling, 6 L Nalophan^TM^ bags were used. Air samples were collected using a vacuum pump in case of point sources, to collect the gas directly into the sampling bag, due to the depression, by preventing the sampled gas contamination [31]. Sampling on area sources (e.g., liquid surface) was carried out using a wind tunnel system, as described in previous papers [32]. The bags sampled according to the procedure described were then directly analysed by TD-GC-MS analysis as described in the next section (Section 2.2).

### 2.2. Chemical Analysis

The chemical analysis of volatile organic compounds (VOCs) was conducted by chromatography/mass spectrometry (GC/MS), coupled with thermal desorption (TD), using a MASTER TOF GC/MS coupled with MASTER TD (DANI Instruments S.p.A., Cologno Monzese, Italy). The air samples were collected directly from the Nalophan™ sampling bags, by using a calibrated pump (DANI Master Air Sampler, DANI Instruments S.p.A., Italy), and then sent to a thermal desorber, by focusing the compounds present in the bags on a packed trap (Trap filled with Tenax^®^ GR, Carbotrap^®^ and Carbosieve SIII, DANI Instruments S.p.A, Cologno Monzese, Italy). After the thermal desorption from the trap, the compounds were injected onto the capillary column (MEGA-VOC 2-0.25 mm × 2.50 μm × 30 m, MEGA s.r.l, Legnano, Italy). This column is developed for the analysis of VOCs mixtures. The carrier gas was helium (99.999%, SAPIO Group, Caponago, Italy) at a flow rate of 0.7 mL/min. A volume of 100 mL of gas was sampled from the bags by a calibrated pump (DANI Master Air Sampler, DANI Instruments S.p.A., Cologno Monzese, Italy), and thermal desorption was carried out and analytes were injected into the capillary column by a transfer line heated at 250 °C. The temperature for the injector was 250 °C. The temperature program started at 45 °C and was raised to 250 °C at a rate of 10 °C/min. Electron impact source was obtained with electron energy of 70 eV. Mass spectral data were acquired over a mass range of 40–200 amu. Quantitative estimation of analytes was conducted by the external standard method, using calibration standards of toluene obtained for dilution of cylinder (SAPIO Group, Caponago, Italy). To obtain the amount of the single compounds, the response factor of toluene was assumed for the all of the observed chemicals. Therefore, the concentration of substances observed is expressed in mg/m^3^ toluene equivalent (semi-quantification). The concentration values obtained were used for the toxicological evaluation of occupational exposure risk for examiners involved in the olfactometric analysis of odorous refinery samples.

### 2.3. Risk Assessment

A standardized approach for risk assessment in dynamic olfactometry was recently proposed [30]. This method was applied in the present study to assess the non-carcinogenic risk and the carcinogenic risk for panellists exposed to real samples collected at different odour sources of a refinery plant. In more detail, the potential non-carcinogenic chronic risk was evaluated using the Hazard Index (HI) [33], which is, in turn, equal to the sum of each chemical component’s Hazard Quotient (Equation (1)):

(1) $$\mathrm{HI}=\overset{N}{\underset{i}{\sum }}{\mathrm{HQ}}_{i}=\overset{N}{\underset{i}{\sum }}\frac{C_{\exp_{i}}}{\mathrm{Occupational}\;\mathrm{Limit}\;\mathrm{Value}}$$

where HQ is the Hazard Quotient to be evaluated for all of the compounds present in the mixture, calculated by dividing the concentration of chemical present in the odour sample (C_exp_) by the exposure reference concentration. In this scenario, the reference concentration is the occupational limit value for the pollutant considered. Focusing on panellists’ exposure, the occupational limit values for short exposure (15-min) are adopted, according to [30]. 

To apply the HI method, reference values for all chemicals need to be available. In this scenario, reference values for the working exposition were considered. In particular, referring to the previous study [30], considering the panel’s work activity and their exposure time to the compounds, the value defined for the short exposure was chosen as OEL. Unfortunately, these values are not available for many compounds. Anyway, a first approach has been established considering, for the toxicological assessment, only the chemicals for which exist an Occupational Limit Value (OEL). Thus, hierarchical criteria, described in [30], have also been proposed and applied here for selecting the most appropriate reference value for each chemical in odour samples between international and national OELs, Derived No Effect Levels (DNELs), and other short-term OELs. If specific OELs are not available, two different approaches are applied to select appropriate generic reference values, based on chemical similarities [30]. When it would not be possible to identify an exposure limit value for a specific compound (and therefore, HQ could not be calculated), a further element of variability in the risk assessment is introduced. 

Therefore, other than the first method (in which only compound-specific OELs were adopted to calculate HQs), a second method was applied: for each compound that does not have an occupational exposure limit value (i.e., when the first method fails), the occupational limit values defined for a family or group of chemical agents (e.g., Hydrocarbons, aliphatic, C6-C8; Hydrocarbons, aliphatic, C9-C14; Hydrocarbons, aromatic, C9-C14; etc.) was applied. These occupational limit values for the family/group of chemicals, reported in GESTIS Database—International Limit Values for Chemical Agents, described the exposure limit for an entire class of compounds, and can, therefore, be used when specific information is lacking. In Table 1, the OELs for the family or group of chemical agents adopted in the risk assessment were reported. These OEL-15 min values defined for family/group are available in GESTIS Database—International Limit Values for Chemical Agents (https://gestis-database.dguv.de/ (accessed on 10 January 2022), and they are defined at the national level. If several OEL values are reported for the same group, the lowest available value has been adopted in order to apply a more precautionary approach.

A third method for defining a limit value for a family or group of chemical agents was applied: this is the case for those chemicals not characterized with an OEL using the 1st method, where the reciprocal calculation procedure (RCP) for deriving exposure limits was applied as described in previous works [34,35]. RCP could be used to derive OELs for refined hydrocarbon solvents based on their composition, strictly following the indications provided by this method. Despite some limitations of this method [35], for this case study, it was considered useful to borrow and adapt the RCP method, applying it only to the fraction of the mixture for components in which it was not possible to find a chemical-specific limit value, thus introducing a simplification and an approximation to the original method. To select the values to be adopted from those available, the lowest one was chosen in order to adopt a precautionary approach.

A comparison of the three approaches proposed to establish an OEL for the compounds not defined in the 1st method (specific OEL) was conducted, and can be seen in the Results and Discussion section (Section 3). 

**Figure 1:**
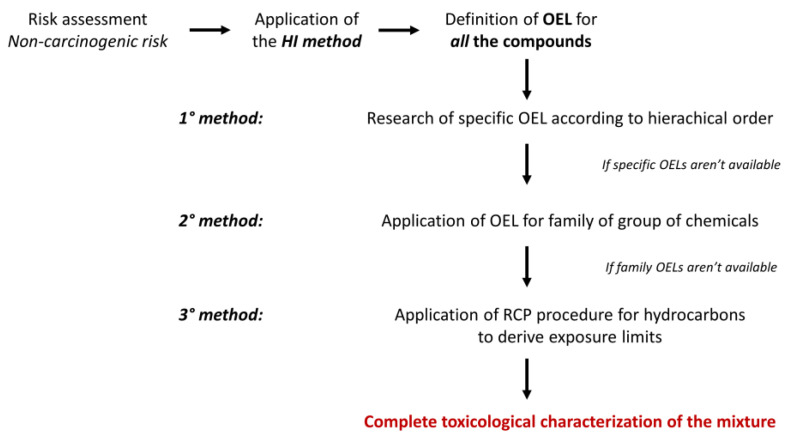
Diagram of OEL definition proposed to obtain the complete toxicological characterization.

The entire scheme for the definition of OEL values to be adopted in the risk assessment of non-carcinogenic risk is reported in Figure 1.

The adopted risk assessment method also allows characterising the carcinogenic excess lifetime risk posed by the odour samples. This was based on Equation (2), as reported in the existing guideline [36].

(2) $$\mathrm{Inhalation}\;\mathrm{Risk}=\mathrm{CDI}\;\times \mathrm{IUR}=\frac{C_{\mathrm{air}}\times {\mathrm{EF}}_{\mathrm{iw}}\times {\mathrm{ED}}_{\mathrm{iw}}\times {\mathrm{ET}}_{\mathrm{iw}}\;}{{\mathrm{AT}}_{\mathrm{iw}}\times \mathrm{LT}}\times \mathrm{IUR}$$

where IUR is the Inhalation Unit Risk, and CDI is the chronic daily intake, expressed in µg/m^3^. IUR values were retrieved by the Risk Assessment Information System (https://rais.ornl.gov/ (accessed on 13 January 2022)); CDI values were calculated in accordance with the working exposure time of the examiners involved in the olfactometric analysis: C_air_ is the concentration observed of the pollutant (µg/m^3^); exposure time (ET_iw_) (hours/day) and exposure frequency (EF_iw_) (day/year) refer to the frequency with which the exposure occurs; exposure duration (ED_iw_) (year) is the amount of time that an individual is exposed to the contaminant. Averaging time (AT_iw_) (days/years) is the amount of time over which exposure is averaged. For carcinogens, the concentration is averaged over the lifetime of the exposed individual (assumed to be 70 years). It is important to remark that in this assessment, the considered concentration of the pollutant is kept constant for the calculation, and set equal to the one measured in each oil refinery sample: in reality, the assessors would be exposed to a multitude of samples, differentiated by types of plant and sampling points. CDI values were calculated based on parameters defined for two different olfactometric laboratories (a “commercial” laboratory owned by a private company and an “institutional” laboratory owned by an environmental inspection authority), derived from a previous study [19] and applied for this study. The adopted data are reported in Table S1. It is important to underline that every laboratory has to evaluate these parameters according to the specific working activity of its own panel. The Inhalation Risk (IR) factor for each carcinogen (i.e., category 1A and 1B) and suspected carcinogen (i.e., category 2) pollutants, following the definition reported in the Regulation (EC) No 1272/2008 [37], in the sample mixture were calculated and were summed to obtain the total Inhalation Risk of the sample. About this last point, it should be noted that in the occupational setting, an acceptable level of risk may be 10^−4^ (1 in 10,000 workers) over a lifetime, due to the intrinsic and accepted risks of the profession, other than due to the fact that workers are subject to risk-management measures (i.e., health surveillance, personal protective equipment, etc.). To recall, technical standards and regulations in the dynamic olfactometry field impose strict limits on the possible protective measures to be taken to ensure the safety of the panellists; in particular, (i) removal of hazardous pollutants are not allowed to avoid alteration of the sample, and (ii) personal protective equipment (i.e., respiratory protective equipment) to protect the panellists against inhalation of hazardous substances cannot be adopted. Thus, for the purpose of this study, concerning cancerogenic risk, community rather than occupational settings criteria were adopted; in this framework, widely accepted criteria consider Lifetime Excess Cancer Risks between 1 in 1,000,000 (10^−6^) and 1 in 100,000 (10^−5^) due to non-occupational exposure being essentially negligible. In more detail, (i) a Lifetime Excess Cancer Risk < 10^−6^ (for a single compound) and <10^−5^ (for mixtures) can be considered negligible. Thus, for this study, an acceptable non-carcinogenic risk level is defined for an HI lower than 1; an acceptable carcinogenic risk level is defined for an Inhalation Risk lower than 10^−6^ for a single compound and lower than 10^−5^ for mixtures. If HI or IR is higher than these parameters, a minimum dilution value must be set to protect panellists’ health [30].

## 3. Results and Discussion

The single substances present in each odorous sample collected at the refinery plant were identified and quantified via TD-GC-MS. For all of the analysed samples, the minimum and maximum observed concentration ranges (C_MIN_–C_MAX_) of each different compound during the campaign are shown in Table S2. These concentrations are expressed in mg/m^3^ of toluene equivalent. Odour samples collected at refinery plants could consist of a huge amount of single compounds. Due to this, for many of the detected chemicals, toxicological data may not be available because there is a lack of information, and therefore, specific studies about the dose–response relationship have not been conducted. Therefore, after the identification and quantitative estimation of the identified compounds, the crucial step of this research was the selection of the reference concentration to be adopted in HI evaluation. To select the occupational exposure limit value for workers’ exposure to being adopted for the present evaluation, different databases have been consulted, according to the hierarchical approach described in a previous study [30]. If an occupational exposure limit (OEL) value for a chemical is not available, the occupational limit concentration for chemical groups, if available, has been considered in the elaboration. By reducing the proportion of components without a specific OEL, a more complete risk characterisation can be conducted. In Table S3, the OEL of the different chemicals observed in the samples, selected and applied to evaluate the HI using the different approaches suggested, are reported. In addition to non-carcinogenic risk, the IUR for all of the compounds defined as carcinogenic, or suspected as carcinogenic following the EU definition, was retrieved to evaluate the carcinogenic risk by calculating the Inhalation Risk (IR) parameter. From the evaluation of the toxicological effects’ correlation with the single pollutant, for all the sources, the health risk correlation with exposure to the entire odorous mix was evaluated by summing, for the non-carcinogenic effects, each HQs to obtain the HI and, for carcinogenic risk, the IR of the single compounds. In Table 2, the resulting HI (Equation (1), method 1) and IR (Equation (2)) values for the different samples collected at the refinery plant are presented. The values above the acceptability criteria are highlighted in bold. 

Discussing the samples analysed, as shown in Table 2, concerning non-carcinogenic risk’s correlation with the exposure to refinery plants during olfactometric analysis, the obtained HI values are always lower than 1: in particular, the HI values calculated are 0.326 ± 0.174 (mean ± standard deviation). Therefore, focusing on non-carcinogenic effects, a minimum dilution value is not required to conduct olfactometric analyses of the presented samples. To further investigate the non-carcinogenic risk and obtain the most complete risk characterization, two further HI values were calculated by adopting, for compounds without a specific OEL, the two approximations described in [30] and summarized in the previous chapter, based on chemical groups and reciprocal calculation procedure (RCP) proposed by ACGIH.

The HI evaluated applying the different approaches and the percentage contribution, established in numeric terms, of compounds without a specific OEL in the total sample are reported in Table 3.

As displayed in Table 3, the HI values calculated using the different methods are comparable (mean HI = 0.326, 0.329, and 0.326, for the application of 1st, 2nd, and 3rd methods, respectively), although there is a general decrease in the percentage contribution of compounds without an exposure limit (i.e., those compounds for which HQ cannot be calculated; average values equal to 7.8%, 2.2%, and 3.2 for the application of 1st, 2nd, and 3rd methods, respectively). In this case, considering the low contributions to risk estimates of the individual compounds without a reference value to the overall composition, for the toxicological assessment, it is possible to limit calculation to the first approach, without using additional considerations.

Discussing the IR values calculated for the samples analysed, a non-negligible carcinogenic risk was calculated for the majority of the samples collected, when considering the “commercial laboratory” scenario. In this scenario, indeed, the carcinogenic risk is generally higher than the acceptability criteria of 10^−5^ for the mixture (2.19 × 10^−5^ ± 1.21 × 10^−5^). When considering the “institutional laboratory” scenario, IR values are far lower (3.96 × 10^−6^ ± 2.18 × 10^−6^), and, overall, do outline an acceptable carcinogenic risk, thus suggesting how the conditions characterising the exposure scenario can modulate the risk. Anyhow, of all compounds observed in the samples and reported in the Supplementary Material (Table S2), the most important contributors to carcinogenic risk for the samples analysed are benzene and naphthalene: these two chemicals are, among the pollutants observed, the only ones classified as carcinogenic by European legislation. Therefore, it is necessary to define a minimum dilution value for the samples analysed, in particular for IR values observed for the “commercial laboratory” scenario. In the case of the considered samples, and discussing the specific exposure time considered (“commercial laboratory”), the minimum dilution values to be adopted during the olfactometric analysis to guarantee the examiners’ safety are between 1.01 and 5, as reported in Table 4. The minimum dilution values reported in the table are calculated to reduce the calculated toxicological parameter (e.g., IR or HI) below the limits of acceptability (IR < 10^−5^ and HI < 1) by comparing the parameter considered (in the present case, only by IR—“commercial lab”) with the dilution steps operated by the olfactometer. In this way, defining the dilution step (e.g., the concentration level) not to be exceeded during the panel analysis involved is thus not exposed to an occupational hazard. 

**Figure 2:**
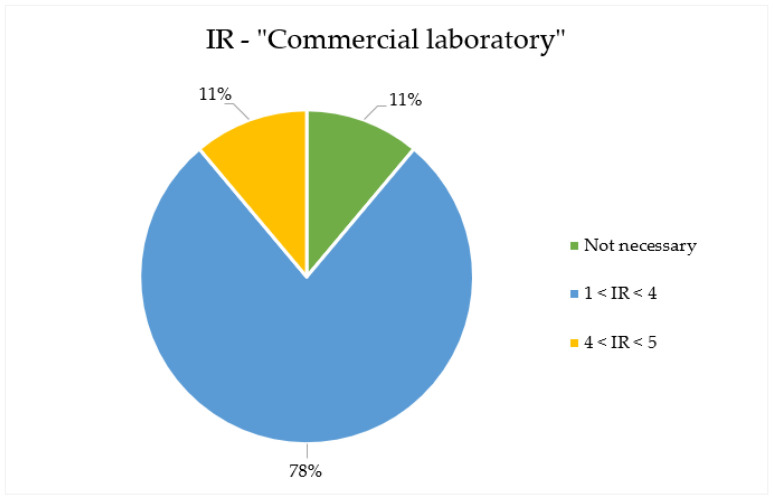
Percentage distribution of samples for IR evaluation (focusing on the “commercial laboratory”).

Focusing on the most critical situation (i.e., the carcinogenic risk evaluated for the “commercial laboratory”), it is important to underline that the IR of the majority of samples analysed (78%) can be reported under the acceptability criteria adopted for lower dilution (between 1 and 4), as reported in Figure 2.

In addition, the odour concentration of these types of refinery samples is generally higher than the minimum dilution observed [38,39]. In addition, it is important to notice that generally, for commercial olfactometers, the minimum dilution factor is 4. For this reason, the odour concentrations measured in these samples, generally, do not require to be specifically diluted, since the safety condition is already met. However, it is essential to emphasise that, though these values represent a first attempt to describe the occupational risk associated with the analysis of refinery samples for examiners involved in dynamic olfactometry, they are only indicative and related to the samples analysed. Indeed, it remains essential, with the help of an expert, to assess individual cases and estimate the minimum dilution factor to be adopted in individual cases. In addition, it is necessary to underline that the risk evaluation, in particular for carcinogenic risk, requests the definition of the specific exposure time, correlated with their working activity, of the panellists involved. This is particularly evident if comparing the results obtained for the carcinogenic risk (IR parameter) obtained for the two different laboratories, where the work activity of the examiners is different in the two scenarios. For this reason, different exposure parameters (Table S1) have to be adopted in the evaluation, and different results are obtained. Therefore, it is necessary to define, for every olfactometric laboratory, the working activity of its group of examiners. For the sake of completeness, it should be noted that some assumptions and limitations must be considered in the interpretation of the results of this case study (the main limitations of the applied method were already reported in [30]). Briefly, HI is a simplified, but conservative approach for calculating non-carcinogenic risk (and which, in this particular application, refers to the risks resulting from short-term exposure). Similarly, the estimated total inhalation carcinogenic risk (i.e., the sum of the inhalation risk factors calculated for each carcinogenic compound in the sample mixture) also implies a strong simplification performed with a conservative approach. Thus, it should be noted that the resulting evaluation is not based on a detailed evaluation of the modes of action and adverse outcome pathways of the hazardous chemicals and carcinogens present in the mixture, and it does not consider chemical interactions as toxicokinetic or toxicodynamic differences. Rather, the proposed approach aimed to provide a simple and flexible evaluation tool, which needs to be rather easy—but conservative enough—to be quickly applied to different samples in the same scenario, to evaluate (in the short-term) the need for risk management measures to allow the performance of dynamic olfactometry tests while protecting the health of the panellists (i.e., to calculate the minimum dilution values of samples). More broadly, the application of this assessment method should be seen a first-tier risk assessment in a wider tiered approach. The application of higher-tier (i.e., more accurate, low-uncertainty) methods must be applied by experts in the field of toxicology, if (i) decidedly non-negligible risks are highlighted by means of the tier-one assessment phase, or (ii) whenever specific in-depth toxicological and carcinogenic risk assessments are required, and (iii) in any case of doubt in the application of the above method.

## 4. Conclusions

The evaluation of occupational risk for examiners involved in dynamic olfactometry is a fundamental step to conducting olfactometric analysis in safe conditions. Among the various industrial activities involved in odour monitoring, refineries are one of the most important. This study aims to apply a new method for the evaluation of occupational risk for panellists involved in olfactometric analyses of refinery emissions based on a real case study. The adopted method aims to solve some of the most critical aspects present in the regulation and the scientific literature available, to obtain the most complete toxicological characterization of the occupational risk. To carry out this evaluation, real samples collected at the main odorous sources of a refinery were analysed by TD-GC-MS analysis to obtain the characterisation and the quantitative estimation of the odorous emissions. It is necessary to highlight that, due to the heterogeneity of the samples and the intention to conduct an untargeted analysis, a semi-quantification was obtained using toluene, due to the complexity of odour, characterised by a great variability of unknown compounds. As a result, it is particularly complex to quantify every single compound present by means of an appropriate calibration line. However, considering the limitations, this analysis, although it represents a first attempt to quantify the compounds potentially inhaled by olfactometric examiners, could be useful as an initial screening to better investigate and characterise the compounds present in odorous emissions. 

After the collection of quantitative and qualitative information about the chemicals present in the emissions, a toxicological method was applied to all of the samples to calculate the non-carcinogenic and carcinogenic risk associated with exposure during olfactometric analysis with a simple, conservative, and flexible evaluation method, to rapidly evaluate the need for risk-management measures to allow the performance of dynamic olfactometry tests while protecting the health of the panellists (i.e., to calculate the minimum dilution values of samples). From this evaluation, according to the exposure time considered in our elaboration, based on the exposure data available in a previous study, it appeared that a non-negligible carcinogenic risk may exist for panellists exposed to the majority of samples. Therefore, to guarantee panellists’ safety, at the exposure conditions considered in our study, a minimum dilution value between 1.01 and 5, according to the specific sample, must be set during the analysis. The minimum dilution values calculated in this study, however, are lower than the general odour concentration of refinery samples. Therefore, this evaluation does not reveal any particular criticalities for panellists’ health during the olfactometric analysis of the refinery odorous samples considered. Some assumptions and limits must be considered in interpreting this study’s results. Although the samples were chosen to reflect a general refinery scenario, specific samples should be selected according to the plant’s contingent requirements (i.e., the existence of particular odorous emissions/particular industrial processes). Indeed, due to the sample size and the very high heterogeneity expected in this type of samples, this study can be then considered as an exploratory, rather than a confirmatory, study. Therefore, the samples evaluated in this study may not directly correspond to similar scenarios elsewhere, and can only be considered representative of emissions from a generic refinery plant. For this reason, it is necessary to emphasise that the evaluations carried out here are a first attempt to provide an assessment of the potential exposure risk for olfactometric workers exposed to refinery odour samples. To evaluate the particular risk associated with specific refinery emissions, it will be necessary to evaluate on a case-by-case basis, investigating the chemical nature of the sample analysed, and evaluate, according to the method proposed and the solutions described in this paper, the occupational risk correlated with other refinery samples. 

## Figures and Tables

**Table 1:** ELs adopted in the 2nd method for family/group of chemicals.

OELs for Groups of Chemicals Adopted in the 2nd Method		
Family or Group of Chemicals	OEL-15 min [mg/m^3^]	Country
Hydrocarbons, aliphatic, C6–C8	1400	Germany (AGS)
Hydrocarbons, aliphatic, C9–C14	600	Germany (AGS)
Hydrocarbon mixtures, aliphatic C5–C8	300	Latvia
Hydrocarbons, aromatic, C9–C14	100	Germany (AGS)
Hydrocarbon mixtures, aromatic C7–C8	300	Latvia
Petroleum, industrial-heptane type	1200	Sweden
Petroleum, industrial-hexane type	250	Sweden
Petroleum, industrial-octane type	1400	Sweden

**Table 2:** Risk assessment evaluation for refineries samples. HI = hazard index; IR = inhalation risk.

Area/Location		N° of Samples	Non-Carcinogenic Risk	Carcinogenic Risk	
			HI	IR (Commercial Lab)	IR (Institutional Lab)
Wastewater treatment	Wastewater collection tank	2	0.227	**1.78 × 10^−5^**	3.22 × 10^−6^
			0.152	9.38 × 10^−6^	1.70 × 10^−6^
	Oil–water separation tank	1	0.174	**1.17 × 10^−5^**	2.11 × 10^−6^
	Flocculation tank	2	0.383	**2.52 × 10^−5^**	4,57 × 10^−6^
			0.149	**1.01 × 10^−5^**	1.83 × 10^−6^
	Flotation tank	2	0.418	**2.80 × 10^−5^**	5.07 × 10^−6^
			0.164	**1.07 × 10^−5^**	1.94 × 10^−6^
	Oily sludge tank	1	0.287	**1.84 × 10^−5^**	3.33 × 10^−6^
	Activated-sludge treatment tank	2	0.794	**5.00 × 10^−5^**	9.05 × 10^−6^
			0.349	**2.49 × 10^−5^**	4.51 × 10^−6^
	Sedimentation tank	2	0.290	**2.01 × 10^−5^**	3.64 × 10^−6^
			0.618	**4.52 × 10^−5^**	8.18 × 10^−6^
	Sludge thickener tank	1	0.382	**2.77 × 10^−5^**	5,01 × 10^−6^
	Final collection	1	0.484	**3.51 × 10^−5^**	6.35 × 10^−6^
Vapour recovery unit system	Vapour recovery unit outlet	3	0.325	**2.22 × 10^−5^**	4.02 × 10^−6^
			0.278	**1.32 × 10^−5^**	2.39 × 10^−6^
			0.258	**1.62 × 10^−5^**	2.94 × 10^−6^
Tanks	Fuel oil tank	1	0.128	7.51 × 10^−6^	1.36 × 10^−6^
Mean (±standard deviation)		18	0.326 (±0.174)	2.19 × 10^−5^ (±1.21 × 10^−5^)	3.96 × 10^−6^ (±2.18 × 10^−6^)
Median (min; max)			0.288 (0.128; 0.794)	1.93 × 10^−5^ (7.51 × 10^−6^; 5.00 × 10^−5^)	3.49 × 10^−6^ (1.36 × 10^−6^; 9.05 × 10^−6^)

The HI and IR values above the acceptability criteria in bold.

**Table 3:** Non-carcinogenic risk evaluated applying different approaches: HI = hazard index, calculated with different methods: 1st method (compound-specific OEL); 2nd method (1st method + OELs for groups of chemicals); 3rd method (1st method + RCP). N.C. (%) = percentage of the detected chemical compounds for which it was not possible to calculate a HQ value.

Area/Location		N° of Sample	1st Method (Compound-Specific OEL)		2nd Method (1st Method + OELs for Groups of Chemicals)		3rd Method (1st Method + RCP Method)	
			HI	N.C. (%)	HI	N.C. (%)	HI	N.C. (%)
Wastewater treatment	Wastewater collection tank	2	0.227	35.0%	0.248	4.5%	0.230	4.5%
			0.152	1.2%	0.152	1.2%	0.152	1.2%
	Oil–water separation tank	1	0.174	5.7%	0.175	0.4%	0.174	0.4%
	Flocculation tank	2	0.383	5.0%	0.385	1.5%	0.383	2.3%
			0.149	6.2%	0.150	1.0%	0.149	2.0%
	Flotation tank	2	0.418	3.2%	0.418	1.5%	0.418	2.7%
			0.164	5.2%	0.165	1.4%	0.164	2.5%
	Oily sludge tank	1	0.287	4.5%	0.287	1.8%	0.287	1.8%
	Activated-sludge treatment tank	2	0.794	2.6%	0.794	0.3%	0.794	2.2%
			0.349	3.5%	0.349	1.3%	0.349	2.8%
	Sedimentation tank	2	0.290	24.8%	0.292	19.3%	0.290	20.2%
			0.618	3.2%	0.619	0.9%	0.618	2.5%
	Sludge thickener tank	1	0.382	4.5%	0.383	1.5%	0.382	2.7%
	Final collection	1	0.484	2.7%	0.485	1.1%	0.484	2.4%
Vapour recovery unit system	Vapour recovery unit outlet	3	0.325	11.1%	0.329	1.5%	0.325	4.1%
			0.278	3.5%	0.298	0.1%	0.278	0.1%
			0.258	6.4%	0.261	0.7%	0.259	1.5%
Tanks	Fuel oil tank	1	0.128	2.5%	0.131	0.4%	0.128	0.9%
Mean (±standard deviation)		18	0.326 (±0.174)	7.8 (±8.7)	0.329 (±0.173)	2.2 (±4.4)	0.326 (±0.174)	3.2 (±4.4)
Median (min; max)			0.288 (0.128; 0.794)	4.5. (1.2; 35.0)	0.295 (0.131; 0.794)	1.2. (0.1; 19.3)	0.288 (0.128; 0.794)	2.4 (0.1; 20.2)

**Table 4:** Minimum dilution values for the samples considered. If a sample respects the acceptability criteria, a minimum dilution value is not necessary.

Area/Location		N° of Samples	Minimum Dilution Value		
			HI	IR (Commercial Lab)	IR (Institutional Lab)
Wastewater treatment	Wastewater collection tank	2	Not necessary	1.78	Not necessary
			Not necessary	Not necessary	Not necessary
	Oil–water separation tank	1	Not necessary	1.17	Not necessary
	Flocculation tank	2	Not necessary	2.52	Not necessary
			Not necessary	1.01	Not necessary
	Flotation tank	2	Not necessary	2.80	Not necessary
			Not necessary	1.07	Not necessary
	Oily sludge tank	1	Not necessary	1.84	Not necessary
	Activated-sludge treatment tank	2	Not necessary	5.00	Not necessary
			Not necessary	2.49	Not necessary
	Sedimentation tank	2	Not necessary	2.01	Not necessary
			Not necessary	4.52	Not necessary
	Sludge thickener tank	1	Not necessary	2.77	Not necessary
	Final collection	1	Not necessary	3.51	Not necessary
Vapour recovery unit system	Vapour recovery unit outlet	3	Not necessary	2.22	Not necessary
			Not necessary	1.32	Not necessary
			Not necessary	1.62	Not necessary
Tanks	Fuel oil tank	1	Not necessary	Not necessary	Not necessary

## Data Availability

Not applicable.

## Supplementary Materials

The following supporting information can be downloaded at: https://www.mdpi.com/article/10.3390/toxics10050202/s1, Table S1: Parameter for CDI calculation; Table S2: Concentration of pollutants observed in the samples collected. The quantification of the single compounds was performed assuming a unit response factor for the different chemicals observed based on toluene calibration curve; Table S3: Pollutants observed in the samples analysed, with their CAS number and the OEL (expressed in mg/m^3^), applied to evaluate the non-carcinogenic risk.
