# Supplementary material for: Dynamic Olfactometry and Oil Refinery Odour Samples: Application of a New Method for Occupational Risk Assessment
Source: Toxics. 2022 Apr 20;10(5):202. doi: 10.3390/toxics10050202 (PMC9144706; doi:10.3390/toxics10050202)
Supplement: Supplementary file 1 [file toxics-10-00202-s001.zip › toxics-1652303-supplementary.pdf]

toxics MDPI

Supplementary Materials: Dynamic Olfactometry and Oil Refinery Odour Samples: Application of a New Method for Occupational Risk Assessment

Andrea Spinazzè, Elisa Polvara, Andrea Cattaneo, Marzio Invernizzi, Domenico Maria Cavallo and Selena Sironi

Table S1. Parameter for CDI calculation.

Parameter [unit]	"Commercial Lab" Scenario	"Institutional Lab" scenario
Inhalation Unit Risk - IUR [μg/m³]	Retrieved by the Risk Assessment Information System (https://rais.ornl.gov/) for carcinogen chemical in the odour sample (Carcinogen cat. 1A, 1B, 2)	
Exposure concentration - C_{air} [μg/m³]	Chemical's concentration in the odour sample (Carcinogen cat. 1A, 1B, 2)	
Exposure Frequency - EF_{iw} [day/year]	90	10
Exposure Duration - ED_{iw} [year]	10	7
Exposure Time - ET_{iw} [hours/day]	0,073	0,17
Averaging time - AT_{iw} [days/years]	365	365
Lifetime of the exposed individuals - LT [years]	70	70

Parameter for CDI calculation was derived from [19] and applied as described in [30].

Table S2. Concentration of pollutants (minimum – maximum) observed in the samples collected. The quantification of the single compounds was performed assuming a unit response factor for the different chemicals observed based on toluene calibration curve.

Compound	$C_{MIN\ tol.\ eq.}$ [mg/m³]	$C_{MAX\ tol.\ eq.}$ [mg/m³]
1,3-Dioxolane, 2-methyl-	0.02	0.17
1-Heptene, 5-methyl-	0.01	0.01
1-Hexanol, 2-ethyl-	0.01	0.01
1-Pentene, 2-methyl-	0.003	0.00
1-Undecanol	0.03	0.71
2,4-Dimethylstyrene	0.02	0.05
2-Butanone	0.07	0.07
2-Methyl-1-butene	0.08	0.08
2-Pentene	0.13	0.13
2-Pentene, (Z)-	0.04	0.04
2-Propanol, 2-methyl-	0.32	0.32
2-Undecene, 6-methyl-,	0.02	0.02
3-Heptene, 3-ethyl-	0.01	0.01
3-Hexene, 3-methyl-,	0.06	0.06
3-Methyl-3-hexene	0.02	0.02
4-Decene	0.06	0.06
Benzene	0.24	1.83
Benzene, (1,1-dimethylpropyl)-	0.04	0.04
Benzene, (1-methylethyl)-	0.004	0.52
Benzene, (1-methylpropyl)-	0.01	0.24
Benzene, (2-methyloctyl)-	0.01	0.02
Benzene, (3,3-dimethylbutyl)-	0.05	0.05
Benzene, 1,2,3-trimethyl-	0.004	0.55
Benzene, 1,2,4,5-tetramethyl-	0.03	0.03

Compound	$C_{MIN\ tol.\ eq.}$ [mg/m^3]	$C_{MAX\ tol.\ eq.}$ [mg/m^3]
Benzene, 1,2,4-trimethyl-	0.01	0.01
Benzene, 1,2-diethyl-	0.03	0.03
Benzene, 1,3-diethyl-	0.004	0.07
Benzene, 1,4-diethyl-	0.01	0.03
Benzene, 1-ethenyl-2-methyl-	0.01	0.01
Benzene, 1-ethyl-2-methyl-	0.02	0.07
Benzene, 1-ethyl-3-methyl-	0.01	0.06
Benzene, 1-ethyl-4-methyl-	0.005	0.13
Benzene, 1-methyl-3-(1-methylethyl)-	0.01	0.05
Benzene, 1-methyl-3-propyl-	0.02	0.02
Benzene, 1-methyl-4-(1-methylpropyl)-	0.03	0.03
Benzene, 1-methyl-4-propyl-	0.01	0.29
Benzene, 1-propynyl-	0.01	0.01
Benzene, 4-ethyl-1,2-dimethyl-	0.00	0.005
Benzene, propyl-	0.01	0.01
Butanal, 2-methyl-	0.03	0.03
Butane	10.7	41.4
Butane, 2,2,3,3-tetramethyl-	4.89	4.89
Butane, 2-methyl-	0.01	0.21
cis-3-Decene	0.02	0.05
cis-4-Decene	0.03	0.09
Cycloheptane, methyl-	0.005	0.005
Cyclohexane, 1,1,3-trimethyl-	0.01	0.01
Cyclohexane, 1,1-dimethyl-2-propyl-	0.03	0.03
Cyclohexane, 1,2-dimethyl-	0.004	0.01
Cyclohexane, 1,3-dimethyl-	0.01	0.43
Cyclohexane, 1,3-dimethyl-, cis-	0.01	0.12
Cyclohexane, 1,3-dimethyl-, trans-	0.09	0.09
Cyclohexane, 1-ethyl-1-methyl-	0.16	0.16
Cyclohexane, 1-ethyl-2,4-dimethyl-	0.41	0.41
Cyclohexane, 1-methyl-3-propyl-	0.17	0.17
Cyclohexane, butyl-	0.14	0.14
Cyclohexane, ethyl-	0.01	0.41
Cyclohexane, methyl-	0.004	1.03
Cyclopentane	0.01	1.89
Cyclopentane, 1,2,3-trimethyl-	0.003	0.003
Cyclopentane, 1,2,3-trimethyl-,	0.11	0.11
Cyclopentane, 1,2,4-trimethyl-	0.06	0.09
Cyclopentane, 1-ethyl-3-methyl-	0.14	0.14
Cyclopentane, ethyl-	0.003	0.08
Cyclopentane, methyl-	0.003	0.14
Cyclopentane, propyl-	0.01	0.08
Cyclopropane, 1,2-dimethyl-	5.04	5.04
Decane	0.07	2.47
Decane, 2,5,9-trimethyl-	0.01	0.01
Decane, 3,7-dimethyl-	0.03	0.03
Decane, 5-methyl-	0.01	0.01
Decanedioic acid, dibutyl ester	1.82	1.82
Dicyclopentadiene	0.02	0.53
Disulfide, dimethyl	0.004	0.01
Dodecane	0.01	0.06
Ethylbenzene	1.49	6.06
Heptane	0.004	1.42

Compound	$C_{MIN\ tol.\ eq.}$ [mg/m³]	$C_{MAX\ tol.\ eq.}$ [mg/m³]
Heptane, 2,3,5-trimethyl-	0.94	0.94
Heptane, 2,3-dimethyl-	0.003	0.21
Heptane, 2,4,6-trimethyl-	0.003	0.06
Heptane, 2,4-dimethyl-	0.01	0.04
Heptane, 2,5-dimethyl-	0.003	0.20
Heptane, 2,6-dimethyl-	0.01	0.27
Heptane, 2-methyl-	0.003	1.06
Heptane, 3-ethyl-2-methyl-	0.03	0.03
Heptane, 3-methyl-	0.01	0.79
Heptane, 4-ethyl-	0.06	0.06
Hexadecane	0.01	0.01
Hexane, 2,2,5-trimethyl-	0.67	0.67
Hexane, 2,2-dimethyl-	0.03	0.03
Hexane, 2,4-dimethyl-	0.02	2.08
Hexane, 2,5-dimethyl-	0.77	0.77
Hexane, 2-methyl-	0.01	0.95
Hexane, 3,4-dimethyl-	0.01	0.01
Hexane, 3-ethyl-	0.11	0.11
Hexane, 3-methyl-	0.01	1.07
Indene	0.01	0.01
Mesitylene	0.01	0.12
Myrtenyl 2-methyl butyrate	0.03	0.03
Naphthalene	0.03	0.19
Naphthalene, 1,2,3,4-tetrahydro-	0.004	0.004
n-Hexane	0.003	0.37
Nonanal	0.01	0.01
Nonane	0.005	2.22
Nonane, 2,6-dimethyl-	0.01	0.02
Nonane, 3,7-dimethyl-	0.08	0.08
Nonane, 3-methyl-	0.01	0.37
Nonane, 4-methyl-	0.05	0.07
Octane	0.01	0.17
Octane, 2,3,6-trimethyl-	0.02	0.35
Octane, 2,3,7-trimethyl-	0.01	0.01
Octane, 2,3-dimethyl-	0.02	0.02
Octane, 2,6-dimethyl-	0.003	0.004
Octane, 2-methyl-	0.01	0.05
Octane, 3,6-dimethyl-	0.02	0.53
Octane, 3-methyl-	0.50	0.50
Octane, 4-methyl-	0.003	0.72
o-Cymene	0.01	0.05
o-Xylene	0.07	0.42
p-Cymene	0.01	0.05
Pentane	0.01	109.9
Pentane, 2,2,3-trimethyl-	0.20	0.20
Pentane, 2,2,4,4-tetramethyl-	0.06	0.06
Pentane, 2,3,3-trimethyl-	0.04	3.94
Pentane, 2,3,4-trimethyl-	0.02	2.27
Pentane, 2,4-dimethyl-	0.08	0.08
Pentane, 2-methyl-	0.002	0.14
Pentane, 3-ethyl-2,2-dimethyl-	0.02	0.08
Pentane, 3-methyl-	0.003	0.10
Propane	0.07	0.60

**Compound**	**CMIN tol. eq. [mg/m³]**	**CMAX tol. eq. [mg/m³]**
Propane, 2-ethoxy-2-methyl-	0.005	0.03
p-Xylene	0.11	1.73
Styrene	0.002	0.02
Sulfurous acid, cyclohexylmethyl undecyl ester	0.35	0.35
Toluene	0.02	1.20
trans-3-Decene	0.01	0.06
trans-4-Decene	0.02	0.20
Tridecane	0.01	0.02
Undecane	0.01	0.70
Undecane, 2,6-dimethyl-	0.01	0.03

Table S3. Pollutants observed in the samples analysed, with their CAS number and the OEL (expressed in mg/m³), applied to evaluate the non-carcinogenic risk.

**Compound**	**CAS**	**1st method compound-specific OEL**	**2nd method 1st method + OELs for groups of chemicals**	**3rd method 1st method+ RCP method**	**OEL category only compound-specific**
1,3-Dioxolane, 2-methyl-	497-26-7	n.d.	n.d.	n.d.	n.d.
1-Heptene, 5-methyl-	13151-04-7	n.d.	300	n.d.	n.d.
1-Hexanol, 2-ethyl-	104-76-7	10.8	10.8	10.8	Switzerland
1-Pentene, 2-methyl-	763-29-1	n.d.	300	n.d.	n.d.
1-Undecanol	112-42-5	n.d.	n.d.	n.d.	n.d.
2,4-Dimethylstyrene	2234-20-0	n.d.	100	100	n.d.
2-Butanone	78-93-3	900	900	900	EU OEL
2-Methyl-1-butene	513-35-9	n.d.	300	n.d.	n.d.
2-Pentene	109-68-2	n.d.	300	n.d.	n.d.
2-Pentene, (Z)-	627-20-3	n.d.	300	n.d.	n.d.
2-Propanol, 2-methyl-	75-65-0	214	214	214	DNEL W - ST systemic
2-Undecene, 6-methyl-, (Z)-	74630-43-6	n.d.	600	n.d.	n.d.
3-Heptene, 3-ethyl-	74764-46-8	n.d.	600	n.d.	n.d.
3-Hexene, 3-methyl-, (E)-	3899-36-3	n.d.	300	n.d.	n.d.
3-Methyl-3-hexene	3404-65-7	n.d.	300	n.d.	n.d.
4-Decene	19398-88-0	n.d.	600	n.d.	n.d.
Benzene	71-43-2	3.25	3.25	3.25	EU OEL
Benzene, (1,1-dimethylpropyl)-	2049-95-8	n.d.	100	100	n.d.
Benzene, (1-methylethyl)-	98-82-8	250	250	250	EU OEL
Benzene, (1-methylpropyl)-	135-98-8	n.d.	100	100	n.d.
Benzene, (2-methyloctyl)-	49826-80-4	n.d.	n.d.	100	n.d.
Benzene, (3,3-dimethylbutyl)-	17314-92-0	n.d.	100	100	n.d.
Benzene, 1,2,3-trimethyl-	526-73-8	170	170	170	Poland
Benzene, 1,2,4,5-tetramethyl-	95-93-2	n.d.	100	100	n.d.
Benzene, 1,2,4-trimethyl-	95-63-6	100	100	100	DNEL W - ST systemic
Benzene, 1,2-diethyl-	135-01-3	44.8	44.8	44.8	Germany (AGS)
Benzene, 1,3-diethyl-	141-93-5	22	22	22	Germany (AGS)
Benzene, 1,4-diethyl-	105-05-5	8.82	8.82	8.82	DNEL W - ST systemic
Benzene, 1-ethenyl-2-methyl-	611-15-4	196	196	196	Germany (DFG)
Benzene, 1-ethyl-2-methyl-	611-14-3	n.d.	100	100	n.d.
Benzene, 1-ethyl-3-methyl-	620-14-4	n.d.	100	100	n.d.
Benzene, 1-ethyl-4-methyl-	622-96-8	400	400	400	Romania
Benzene, 1-methyl-3-(1-methylethyl)-	535-77-3	270	270	270	Denmark
Benzene, 1-methyl-3-propyl-	1074-43-7	n.d.	100	100	n.d.

Benzene, 1-methyl-4-(1-methylpropyl)-	1595-16-0	n.d.	100	100	n.d.
Benzene, 1-methyl-4-propyl-	1074-55-1	n.d.	100	100	n.d.
Benzene, 1-propynyl-	673-32-5	n.d.	100	100	n.d.
Benzene, 4-ethyl-1,2-dimethyl-	934-80-5	n.d.	100	100	n.d.
Benzene, propyl-	103-65-1	n.d.	100	100	n.d.
Butanal, 2-methyl-	96-17-3	n.d.	n.d.	n.d.	n.d.
Butane	106-97-8	1810	1810	1810	UK
Butane, 2,2,3,3-tetramethyl-	594-82-1	1800	1800	1800	Finland
Butane, 2-methyl-	78-78-4	1000	1000	1000	People Republic of China
cis-3-Decene	19398-86-8	n.d.	600	n.d.	n.d.
Cycloheptane, methyl-	4126-78-7	n.d.	300	800	n.d.
Cyclohexane, 1,1,3-trimethyl-	3073-66-3	n.d.	600	800	n.d.
Cyclohexane, 1,1-dimethyl-2-propyl-	81983-71-3	n.d.	600	800	n.d.
Cyclohexane, 1,2-dimethyl-	583-57-3	n.d.	300	800	n.d.
Cyclohexane, 1,3-dimethyl-	591-21-9	n.d.	300	800	n.d.
Cyclohexane, 1,3-dimethyl-, cis-	638-04-0	n.d.	300	800	n.d.
Cyclohexane, 1,3-dimethyl-, trans-	2207-03-6	n.d.	300	800	n.d.
Cyclohexane, 1-ethyl-1-methyl-	4926-90-3	n.d.	600	800	n.d.
Cyclohexane, 1-ethyl-2,4-dimethyl-	61142-69-6	n.d.	600	800	n.d.
Cyclohexane, 1-methyl-3-propyl-	4291-80-9	n.d.	600	800	n.d.
Cyclohexane, butyl-	1678-93-9	n.d.	600	800	n.d.
Cyclohexane, ethyl-	1678-91-7	456	456	456	DNEL W - ST systemic
Cyclohexane, methyl-	108-87-2	1500	1500	1500	Romania
Cyclopentane	287-92-3	1700	1700	1700	Denmark
Cyclopentane, 1,2,3-trimethyl-	2815-57-8	n.d.	300	800	n.d.
Cyclopentane, 1,2,3-trimethyl-	2613-69-6	n.d.	300	800	n.d.
Cyclopentane, 1,2,4-trimethyl-	2815-58-9	n.d.	300	800	n.d.
Cyclopentane, 1-ethyl-3-methyl-	3726-47-4	n.d.	300	800	n.d.
Cyclopentane, ethyl-	1640-89-7	n.d.	300	800	n.d.
Cyclopentane, methyl-	96-37-7	3600	3600	3600	Germany (DFG)
Cyclopentane, propyl-	2040-96-2	n.d.	300	800	n.d.
Cyclopropane, 1,2-dimethyl-, trans-	2402-06-4	n.d.	300	1500	n.d.
Decane	124-18-5	500	500	500	denmark
Decane, 2,5,9-trimethyl-	62108-22-9	n.d.	600	1200	n.d.
Decane, 3,7-dimethyl-	17312-54-8	n.d.	600	1200	n.d.
Decane, 5-methyl-	13151-35-4	n.d.	600	1200	n.d.
Decanedioic acid, dibutyl ester	109-43-3	n.d.	n.d.	n.d.	n.d.
Dicyclopentadiene	77-73-6	2.7	2.7	2.7	Germany (DFG)
Disulfide, dimethyl	624-92-0	15.5	15.5	15.5	DNEL W - ST systemic
Dodecane	112-40-3	n.d.	600	1200	n.d.
Ethylbenzene	100-41-4	884	884	884	EU OEL
Heptane	142-82-5	1200	1200	1200	Sweden
Heptane, 2,3,5-trimethyl-	20278-85-7	n.d.	600	1200	n.d.
Heptane, 2,3-dimethyl-	3074-71-3	n.d.	600	1200	n.d.
Heptane, 2,4,6-trimethyl-	2613-61-8	n.d.	600	1200	n.d.
Heptane, 2,4-dimethyl-	2213-23-2	n.d.	600	1200	n.d.
Heptane, 2,5-dimethyl-	2216-30-0	n.d.	600	1200	n.d.
Heptane, 2,6-dimethyl-	1072-05-5	n.d.	600	1200	n.d.
Heptane, 2-methyl-	592-27-8	n.d.	300	1200	n.d.
Heptane, 3-ethyl-2-methyl-	14676-29-0	n.d.	600	1200	n.d.
Heptane, 3-methyl-	589-81-1	1800	1800	1800	Finland
Heptane, 4-ethyl-	2216-32-2	n.d.	600	1200	n.d.
Hexadecane	544-76-3	n.d.	n.d.	n.d.	n.d.
Hexane, 2,2,5-trimethyl-	3522-94-9	n.d.	600	1200	n.d.

Hexane, 2,2-dimethyl-	590-73-8	18200	18200	18200	Finland
Hexane, 2,4-dimethyl-	589-43-5	1800	1800	1800	Finland
Hexane, 2,5-dimethyl-	592-13-2	1750	1750	1750	Singapore
Hexane, 2-methyl-	591-76-4	2049	2049	2049	TLV-STEL ACGIH
Hexane, 3,4-dimethyl-	583-48-2	1800	1800	1800	Finland
Hexane, 3-ethyl-	619-99-8	1800	1800	1800	Finland
Hexane, 3-methyl-	589-34-4	2049	2049	2049	TLV-STEL ACGIH
Indene	95-13-6	72	72	72	UK
Mesitylene	108-67-8	100	100	100	DNEL W - ST systemic
Myrtenyl 2-methyl butyrate	138530-44-6	n.d.	n.d.	n.d.	n.d.
Naphthalene	91-20-3	8	8	8	Germany (AGS)
Naphthalene, 1,2,3,4-tetrahydro-	119-64-2	11	11	11	Germany (DFG)
n-Hexane	110-54-3	144	144	144	The Netherlands
Nonanal	124-19-6	n.d.	n.d.	n.d.	n.d.
Nonane	111-84-2	1100	1100	1100	Sweden
Nonane, 2,6-dimethyl-	17302-28-2	n.d.	600	1200	n.d.
Nonane, 3,7-dimethyl-	17302-32-8	n.d.	600	1200	n.d.
Nonane, 3-methyl-	59 11-04-6	n.d.	600	1200	n.d.
Nonane, 4-methyl-	17301-94-9	n.d.	600	1200	n.d.
Octane	111-65-9	1400	1400	1400	Sweden
Octane, 2,3,6-trimethyl-	62016-33-5	n.d.	600	1200	n.d.
Octane, 2,3,7-trimethyl-	62016-34-6	n.d.	600	1200	n.d.
Octane, 2,3-dimethyl-	7146-60-3	n.d.	600	1200	n.d.
Octane, 2,6-dimethyl-	2051-30-1	n.d.	600	1200	n.d.
Octane, 2-methyl-	3221-61-2	n.d.	600	1200	n.d.
Octane, 3,6-dimethyl-	15869-94-0	n.d.	600	1200	n.d.
Octane, 3-methyl-	2216-33-3	n.d.	600	1200	n.d.
Octane, 4-methyl-	2216-34-4	n.d.	600	1200	n.d.
o-Cymene	527-84-4	270	270	270	Denmark
o-Xylene	95-47-6	442	442	442	EU OEL
p-Cymene	99-87-6	n.d.	100	100	n.d.
Pentane	109-66-0	1800	1800	1800	USA - NIOSH
Pentane, 2,2,3-trimethyl-	564-02-3	n.d.	600	1200	n.d.
Pentane, 2,2,4,4-tetramethyl-	1070-87-7	n.d.	600	1200	n.d.
Pentane, 2,3,3-trimethyl-	560-21-4	940	940	940	Germany (DFG)
Pentane, 2,3,4-trimethyl-	565-75-3	940	940	940	Germany (DFG)
Pentane, 2,4-dimethyl-	108-08-7	2049	2049	2049	TLV-STEL ACGIH
Pentane, 2-methyl-	107-83-5	1100	1100	1100	Sweden
Pentane, 3-ethyl-2,2-dimethyl-	16747-32-3	n.d.	600	1200	n.d.
Pentane, 3-methyl-	96-14-0	1100	1100	1100	Sweden
Propane	74-98-6	2000	2000	2000	Finland
Propane, 2-ethoxy-2-methyl-	637-92-3	2800	2800	2800	DNEL W - ST systemic
p-Xylene	106-42-3	442	442	442	EU OEL
Styrene	100-42-5	100	100	100	DNEL W - ST systemic
Sulfurous acid, cyclohexylmethyl undecyl ester	959283-66-0	n.d.	n.d.	n.d.	n.d.
Toluene	108-88-3	384	384	384	EU OEL
trans-3-Decene	19150-21-1	n.d.	600	n.d.	n.d.
trans-4-Decene	19398-89-1	n.d.	600	n.d.	n.d.
Tridecane	629-50-5	n.d.	600	1200	n.d.
Undecane	1120-21-4	n.d.	600	1200	n.d.
Undecane, 2,6-dimethyl-	17301-23-4	n.d.	600	1200	n.d.

The OELs were selected and defined from the 3 approaches suggested and described in [30]. The abbreviation "n.d." indicates that an OEL value is not available. In 2nd and 3rd methods, the lowest OEL values for the chemical categories were selected to adopt a precautionary approach.
